# An Essential Difference between the Flavonoids MonoHER and Quercetin in Their Interplay with the Endogenous Antioxidant Network

**DOI:** 10.1371/journal.pone.0013880

**Published:** 2010-11-08

**Authors:** Hilde Jacobs, Mohamed Moalin, Aalt Bast, Wim J. F. van der Vijgh, Guido R. M. M. Haenen

**Affiliations:** 1 Department of Pharmacology and Toxicology, NUTRIM School for Nutrition, Toxicology and Metabolism, Maastricht University Medical Centre (MUMC+), Maastricht, The Netherlands; 2 Faculty of Life Sciences, Hogeschool Zuyd, Heerlen, The Netherlands; University of Wales Bangor, United Kingdom

## Abstract

Antioxidants can scavenge highly reactive radicals. As a result the antioxidants are converted into oxidation products that might cause damage to vital cellular components. To prevent this damage, the human body possesses an intricate network of antioxidants that pass over the reactivity from one antioxidant to another in a controlled way. The aim of the present study was to investigate how the semi-synthetic flavonoid 7-mono-O-(β-hydroxyethyl)-rutoside (monoHER), a potential protective agent against doxorubicin-induced cardiotoxicity, fits into this antioxidant network. This position was compared with that of the well-known flavonoid quercetin. The present study shows that the oxidation products of both monoHER and quercetin are reactive towards thiol groups of both GSH and proteins. However, in human blood plasma, oxidized quercetin easily reacts with protein thiols, whereas oxidized monoHER does not react with plasma protein thiols. Our results indicate that this can be explained by the presence of ascorbate in plasma; ascorbate is able to reduce oxidized monoHER to the parent compound monoHER before oxidized monoHER can react with thiols. This is a major difference with oxidized quercetin that preferentially reacts with thiols rather than ascorbate. The difference in selectivity between monoHER and quercetin originates from an intrinsic difference in the chemical nature of their oxidation products, which was corroborated by molecular quantum chemical calculations. These findings point towards an essential difference between structurally closely related flavonoids in their interplay with the endogenous antioxidant network. The advantage of monoHER is that it can safely channel the reactivity of radicals into the antioxidant network where the reactivity is completely neutralized.

## Introduction

The human body is endowed with a wide range of antioxidants to protect cells from damage induced by free radicals and other reactive species. Glutathione (GSH) is one of the most important endogenous hydrophilic antioxidants [1]. It is synthesized in many different cell types from its constituting amino acids glutamic acid, cysteine and glycine, and is therefore not required in the human diet [1]. The actual antioxidant property of GSH is attributable to the thiol group that is present in its cysteine moiety. As an effective nucleophile, GSH also plays an important role in the protection against electrophilic compounds [2].

Like GSH, ascorbic acid (vitamin C) is also an important hydrophilic antioxidant. In contrast to GSH, ascorbic acid cannot be synthesized by humans and, as a consequence, is required in the human diet [3]. It directly scavenges O_2_^•−^ and ^•^OH and various other radicals.

Phenolic antioxidants comprise α-tocopherol (the most active form of vitamin E) and flavonoids. Like ascorbic acid and most other vitamins, α-tocopherol has to be obtained exclusively from the diet. It is the major lipid-soluble lipoprotein antioxidant [4]. α-Tocopherol is localized in biomembranes and functions as an efficient inhibitor of lipid peroxidation. Flavonoids, on the other hand, are not essential nutrients but they form an integral part of the human diet as they are found in fruits, vegetables, nuts and plant-derived beverages such as tea and wine [5], [6]. They have a wide range of biological activities [7], [8], [9], but are most commonly known for their antioxidant activity. Quercetin is one of the most frequently studied dietary flavonoids [5], [10]. It can scavenge highly reactive species, an activity that is implicated in its health benefits [11], [12].

The flavonoid of interest to us, that closely resembles the chemical structure of quercetin, is the semi-synthetic flavonoid 7-mono-O-(β-hydroxyethyl)-rutoside (monoHER). MonoHER is the most powerful antioxidant constituent of the registered drug Venoruton® [13], which is used in the treatment of chronic venous insufficiency [14]. In vitro screening has shown that monoHER is the most potent protector against cardiotoxicity induced by the anticancer agent doxorubicin within a series of flavonoids [13]. Preclinical experiments have confirmed that monoHER is indeed a potential protective agent against doxorubicin-induced cardiotoxicity [15], [16]. Because of these promising results, clinical trials are being performed to study the protection of intravenously administered monoHER against doxorubicin-induced cardiotoxicity in cancer patients [17], [18]. The antioxidant activity of monoHER is supposed to be involved in its protection. Because of its excellent radical scavenging properties monoHER can effectively protect the heart against free radicals produced by doxorubicin.

During the scavenging of highly reactive species, antioxidants donate an electron or a hydrogen atom to the radical involved, thereby converting the radical into a relatively stable non-radical. In this way the reactivity of the radical is annihilated. However, in this reaction the antioxidant itself is converted into an oxidation product that takes over part of the reactivity of the radical. This oxidized antioxidant might cause damage to vital cellular components [19]. For example, when α-tocopherol scavenges free radicals it is oxidized to produce the corresponding tocopheroxyl radicals [20]. These radicals can recombine with other radicals, such as peroxyl radicals, thereby neutralizing them [21]. However, when these tocopheroxyl radicals cannot be eliminated, lipid peroxidation is aggravated, a phenomenon referred to as tocopherol-mediated peroxidation [21], [22].

To prevent damage by reactive oxidation products of antioxidants, the human body has a refined network of antioxidants that pass over the reactivity from one antioxidant to another in a controlled way, thereby gradually diminishing the reactivity of the radical and recycling the antioxidants. In this way, it has been shown that ascorbate can regenerate α-tocopherol from tocopheroxyl radicals, thereby preventing tocopherol-mediated peroxidation [4], [20], [23]. This illustrates that antioxidants act in synergy to annihilate radicals. Besides preventing damage induced by harmful oxidation products, regeneration is important because it restores the antioxidant network.

The regeneration of α-tocopherol by ascorbate is well documented, however, not much is known on the regeneration of flavonoids. When quercetin protects against free radicals, thiol-reactive oxidation products of quercetin are formed that can cause damage to vital cellular components, a phenomenon known as the quercetin paradox [24]. Recently it was found that the oxidation product of monoHER is also reactive towards thiols [25]. This might have implications for the applicability of monoHER. However, as mentioned above, antioxidants do not act in isolation to protect against oxidative damage.

The aim of the present study was to determine how monoHER fits into the antioxidant network and to get insight in the regeneration of flavonoids. Particularly, the reactivity of oxidized monoHER towards thiols and ascorbate was investigated. In addition a comparison with quercetin was made.

## Materials and Methods

### Ethics Statement

For the study spare, anonymised human blood plasma obtained from the Academic Hospital Maastricht was used according to the procedure approved by the medical ethical review board of the hospital.

### Chemicals

7-mono-O-(β-hydroxyethyl)-rutoside (monoHER) was kindly provided by Novartis Consumer Health (Nyon, Switzerland). Stock solutions of the drug were freshly prepared in a methanol/25 mM phosphate buffer (pH 3.33) mixture (4/1, v/v). Quercetin was purchased from Sigma (St. Louis, MO, USA) and stock solutions were freshly prepared in methanol. Bovine serum albumin (BSA), reduced glutathione (GSH), hydrogen peroxide (H_2_O_2_), horseradish peroxidase (HRP), L-ascorbic acid (vitamin C) and 5,5′-dithiobis-(2-nitrobenzoic acid) (DTNB) were also purchased from Sigma (St. Louis, MO, USA). Trifluoroacetic acid (TFA) was acquired from Sigma-Aldrich (Steinheim, Germany). Acetonitrile HPLC grade and methanol were obtained from Biosolve (Valkenswaard, The Netherlands). 2′-GSH-monoHER was synthesized as described previously [25].

### Oxidation of monoHER

MonoHER was oxidized as described before [25]. Shortly, 50 µM monoHER was incubated for 5 minutes at 37°C together with 1.6 nM HRP and 33 µM H_2_O_2_ in a 145 mM phosphate buffer (pH 7.4). The GSH-monoHER adduct was formed by oxidizing 50 µM monoHER in the presence of 40 µM GSH. To investigate the influence of ascorbate on the oxidation of monoHER and on the formation of the GSH-monoHER adduct, ascorbate (final concentration of 40 µM, unless noted otherwise) was added to the incubation mixtures. The reactions were monitored spectrophotometrically and by HPLC. MonoHER consumption was determined at 355 nm, ascorbate consumption at 270 nm.

### Spectrophotometric analysis

Spectrophotometric analysis was performed with a Varian Carry 50 spectrophotometer (Varian, Mulgrave, VIC, Australia). All absorption spectra were recorded from 220 to 500 nm with a scan speed of 960 nm/min, using quartz cuvettes. The UV/Vis scans were started 30, 150 and 300 seconds after the addition of HRP.

### High-performance liquid chromatography analysis

High-performance liquid chromatography (HPLC) was performed using a HP 1100 series HPLC system (Agilent Technologies, Palo Alto, CA, USA). Analytical separations were achieved using a Supelcosil LC 318 column (5 µm, 25 cm × 4.6 mm) (Supelco, Bellefonte, PA, USA). The mobile phase consisted of water containing 0.1% (v/v) TFA with linear gradients of 5% acetonitrile at t = 0 to 20% acetonitrile at 5 min followed by an increase to 30% acetonitrile at 10 min. Finally 90% acetonitrile was used from 18 min onward for 5 min. The column was reequilibrated with 5% acetonitrile for 5 min. A flow rate of 2 ml/min and an injection volume of 20 µl were used. Detection was carried out with a diode array detector (DAD). The chromatograms presented are based on detection at 355 nm (absorption maximum of monoHER).

### Measurement of thiol reactivity

To determine the thiol reactivity of oxidized monoHER and quercetin, free SH-groups were measured using the DTNB [5, 5′-dithiobis-(2-nitrobenzoic acid)] assay. The incubation mixtures contained 50 µM monoHER, 1.6 nM HRP, 33 µM H_2_O_2_ and 40 µM GSH (or 400 µM BSA) in a 145 mM phosphate buffer (pH 7.4). When the oxidation was performed in the presence of ascorbate, the incubation mixture additionally contained 40 µM of ascorbate. After 0 or 5 minutes of incubation at 37°C, thiol content was measured by adding DTNB (final concentration of 0.6 mM) to the incubation mixtures. The formation of TNB was measured spectrophotometrically at 412 nm. Similar experiments with 50 µM monoHER were performed in human blood plasma to determine the reactivity towards plasma protein thiols. Identical experimental conditions were used to determine thiol reactivity of oxidized quercetin.

### Molecular quantum chemical calculations

Molecular quantum chemical calculations (ab initio level) were performed with the software program Spartan ’06 (Wavefunction, Irvine, CA, USA) to corroborate the experimental results. The Møller Plesset, RI-MP2 with the 6-31G* basis set was used to calculate the relative abundance of the tautomers of oxidized quercetin. The Hartree-Fock method with the 3-21G basis set was used for calculating the equilibrium geometry and the energies of the lowest unoccupied molecular orbital (LUMO) of the quercetin quinone methide and a simplified monoHER quinone (the rutin group at C3-O and the ethoxygroup at C7-O were replaced by methyl groups) and the highest occupied molecular orbital (HOMO) of GSH and ascorbate, unless depicted otherwise. In addition, a LUMO map for the monoHER quinone and the quercetin quinone methide were generated to get a visual on the LUMO distribution.

### Statistics

All experiments were performed, at least, in triplicate. Data are expressed as mean ± SD or as a typical example. Statistical analysis was performed using student's t-test. P values ≤0.05 were considered statistically significant.

## Results

### GSH reacts with oxidized monoHER to form 2′-GSH-monoHER

**Figure 1:**
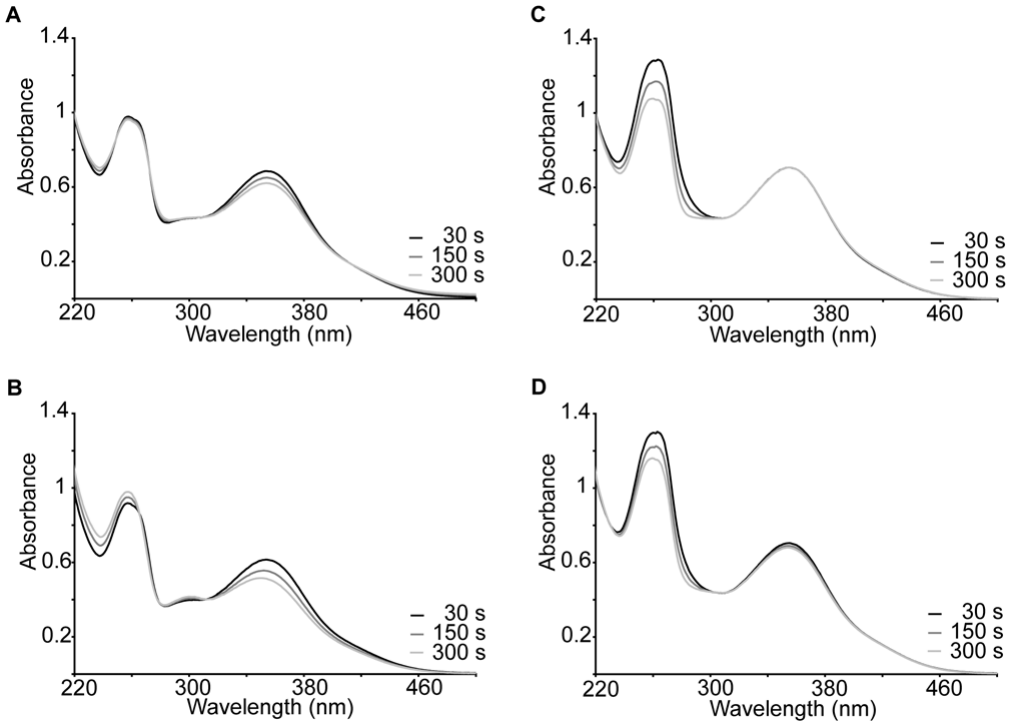
Spectrophotometrical analyses. Spectrophotometrical analysis of the incubation mixture containing (A) 50 µM monoHER, 1.6 nM horseradish peroxidase (HRP) and 33 µM H_2_O_2_. The same experiment was carried out in (B) the presence of 40 µM GSH, (C) 40 µM ascorbate and (D) both 40 µM GSH and 40 µM ascorbate. The UV/Vis scans were recorded 30, 150 and 300 seconds after the addition of HRP. A typical example is shown.

**Figure 2:**
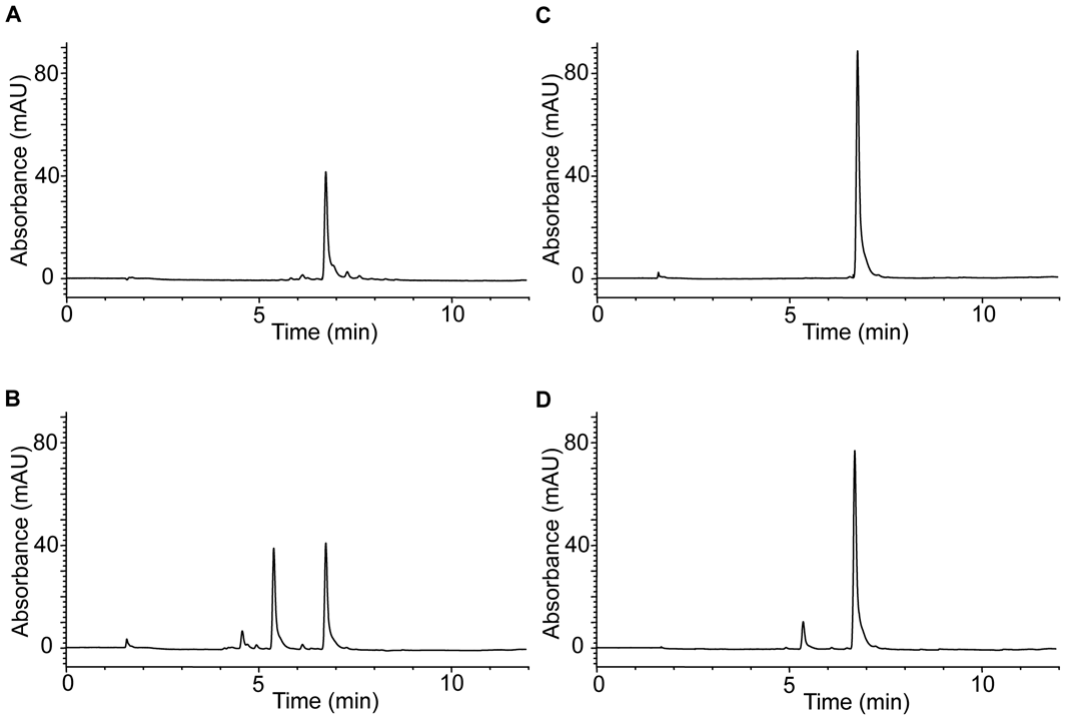
HPLC analyses. HPLC analysis of the incubation mixture containing (A) 50 µM monoHER, 1.6 nM horseradish peroxidase (HRP) and 33 µM H_2_O_2_. The same experiment was carried out in (B) the presence of 40 µM GSH, (C) 40 µM ascorbate and (D) both 40 µM GSH and 40 µM ascorbate. The different incubation mixtures were injected on the HPLC system 5 minutes after the addition of HRP. A typical example is shown. The retention time of monoHER is 6.7 min and that of 2′-GSH-monoHER is 5.4 min. The initial peak height of monoHER before oxidation was 88 mAU, corresponding to a concentration of 50 µM. After 5 min of oxidation the monoHER concentrations in the incubation mixtures A, B, C and D were 22.5 µM, 22.5 µM, 50 µM and 43.5 µM, respectively.

**Figure 3:**
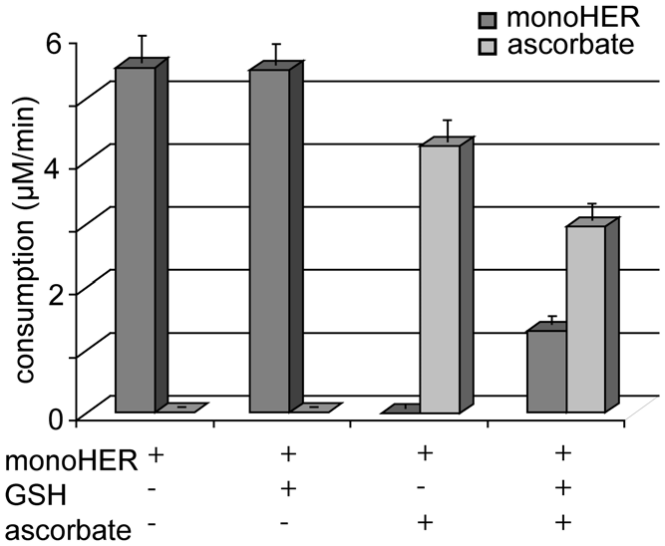
MonoHER and ascorbate consumption rates. Consumption of monoHER and ascorbate in the incubation mixtures containing 50 µM monoHER, 1.6 nM HRP and 33 µM H_2_O_2_ in the presence of either 40 µM GSH, 40 µM ascorbate or both 40 µM GSH and 40 µM ascorbate. The incubation time was 5 minutes. All measurements were carried out in triplicate and data are expressed as mean ± SD.

UV and HPLC analysis (Fig. 1A and Fig. 2A) show that oxidation of 50 µM monoHER by HRP/H_2_O_2_ leads to the consumption of monoHER at a rate of 5.5±0.4 µM/min (Fig. 3). In the presence of 40 µM GSH, all the oxidized monoHER is recovered as 2′-GSH-monoHER at a rate of 5.5±0.3 µM/min (Fig. 3). This is concluded from the appearance of the characteristic UV spectrum of 2′-GSH-monoHER (Fig. 1B) and HPLC analysis of the incubation mixture (Fig. 2B). In the HPLC chromatogram a second peak emerges, eluting at a position identical to that of the synthesized 2′-GSH-monoHER adduct. These data demonstrate that the monoHER quinone is formed as a primary oxidation product and that this oxidation product forms an adduct with GSH.

### Ascorbate reduces oxidized monoHER to monoHER

As shown by UV and HPLC analysis, addition of 40 µM ascorbate to the incubation mixture containing monoHER and HRP/H_2_O_2_ prevents monoHER consumption (Fig. 1C and Fig. 2C). At the same time the ascorbate concentration decreases, as seen in the spectrum as a decrease of the absorption at 270 nm. When monoHER is omitted from the incubation mixture, there is no detectable ascorbate consumption. These findings suggest that monoHER is regenerated from its oxidation product by ascorbate.

The average rate of ascorbate consumption in the presence of monoHER (4.3±0.3 µM/min) is 23% less than monoHER consumption in the absence of ascorbate (5.5±0.4 µM/min) (Fig. 3). Addition of more ascorbate (final concentration of 100 µM) to the incubation mixture reduces the ascorbate consumption to 1.5±0.1 µM/min, 72% less than monoHER consumption without ascorbate. This indicates that the enzyme HRP is also partially inhibited by ascorbate, as has been shown previously [26]. The extent of inhibition depends on the ascorbate concentration.

### Competition between GSH and ascorbate for oxidized monoHER

To investigate the competition between GSH and ascorbate, monoHER was oxidized in the presence of both compounds (Fig. 1D and Fig. 2D). Comparison of the rate of 2′-GSH-monoHER formation (1.3±0.1 µM/min) and the rate of ascorbate consumption (3.0±0.3 µM/min) (Fig. 3) indicates that oxidized monoHER reacts two to three times faster with ascorbate than with GSH. These results are in contrast with those found for quercetin. In a comparable competition experiment with quercetin it was found that oxidized quercetin predominantly reacts with GSH [26].

**Figure 4:**
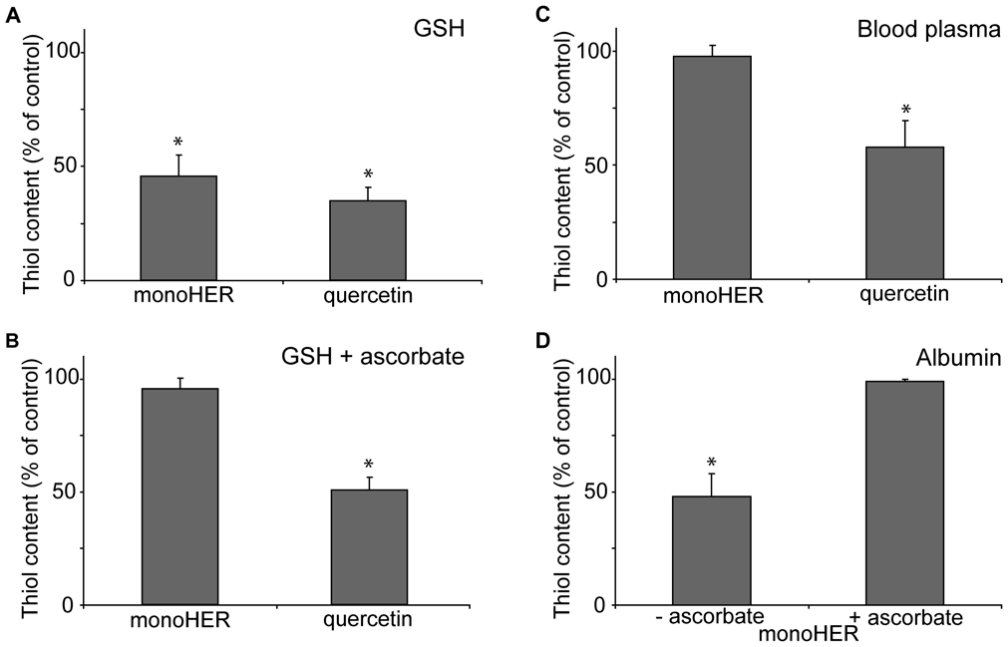
Reactivity of oxidized monoHER and oxidized quercetin towards thiols. Thiol content of the incubation mixture containing 50 µM monoHER or quercetin, 1.6 nM HRP and 33 µM H_2_O_2_ in the presence of either (A) 40 µM GSH, (B) both 40 µM GSH and 40 µM ascorbate, (C) human blood plasma or (D) 400 µM albumin (BSA) (with or without 40 µM ascorbate). The thiol content of the different incubation mixtures was measured 5 minutes after the addition of HRP. All measurements were carried out in triplicate and data are expressed as mean ± SD. *P<0.05 compared to control.

As shown in Fig. 4A, both oxidized monoHER and oxidized quercetin, produced *in situ* by HRP/H_2_O_2_-mediated oxidation, decrease the thiol content of the incubation mixture containing 40 µM GSH to approximately 50% in 5 minutes. The presence of ascorbate (40 µM) does not affect the consumption of thiols by oxidized quercetin (Fig. 4B). In contrast, ascorbate significantly decreases the thiol consumption induced by oxidized monoHER (Fig. 4B). This confirms that oxidized monoHER preferentially reacts with ascorbate, whereas oxidized quercetin preferentially reacts with GSH.

### Competition between protein thiols and ascorbate for oxidized monoHER and oxidized quercetin

Next, monoHER and quercetin were oxidized in human blood plasma. In human blood plasma GSH is practically absent and ascorbate concentrations are 40–60 µM [27]. The generation of oxidized quercetin decreases the thiol content of plasma, i.e. protein thiols, by approximately 40% (Fig. 4C). In contrast, the generation of oxidized monoHER has no effect on the thiol content of human blood plasma (Fig. 4C). An additional experiment shows that oxidized monoHER is able to react with the thiol group of albumin, which is the most abundant plasma protein (Fig. 4D). Ascorbate is able to prevent the reaction of oxidized monoHER with albumin (Fig. 4D).

These results point towards an essential difference between monoHER and quercetin, i.e. oxidized monoHER rather reacts with ascorbate than with protein thiols, while oxidized quercetin preferentially reacts with protein thiols.

### Explanation of the difference in reactivity between oxidized monoHER and oxidized quercetin

**Figure 5:**
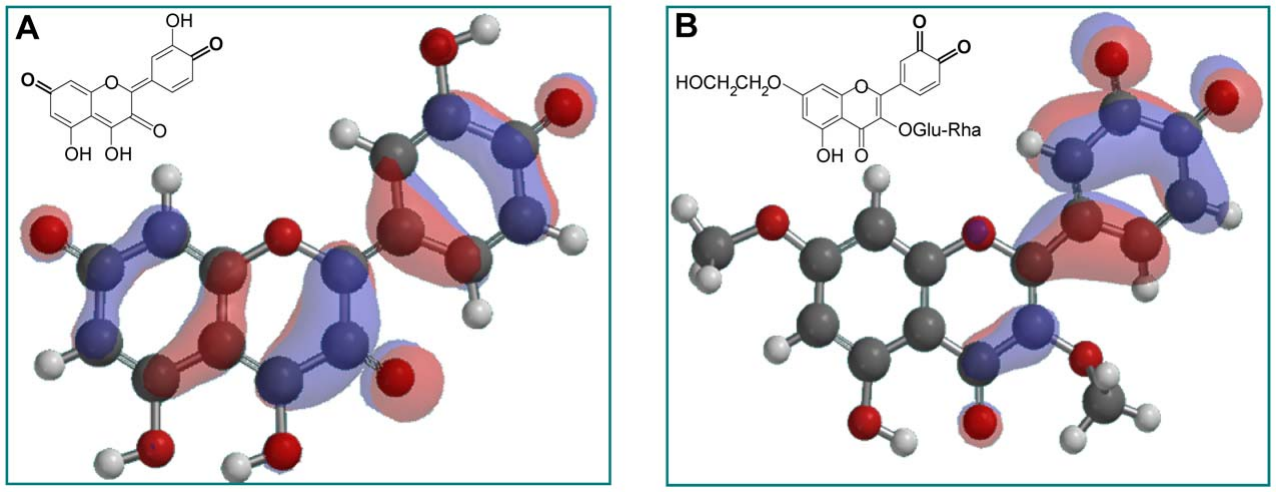
LUMO delocalization maps. LUMO delocalization map of (A) oxidized quercetin and (B) oxidized monoHER. In oxidized monoHER, the rutin group at C3-O and the ethoxygroup at C7-O were replaced by methyl groups and for quercetin, the most abundant tautomer (>99%) was used. The LUMO of oxidized monoHER is localized in the B ring and part of the C ring, while the LUMO of oxidized quercetin is distributed over all the phenolic rings (rings A, C and B).

Molecular quantum chemical calculations show that the tautomer depicted in Fig. 5A, a quinone methide, represents more than 99% of oxidized quercetin. In oxidized monoHER only the ortho-quinone, illustrated in Fig. 5B, can be formed. Generation of a LUMO map of oxidized monoHER and oxidized quercetin shows that the LUMO of oxidized monoHER is restricted to the B ring and part of the C ring, while the LUMO of oxidized quercetin is delocalized over all the phenolic rings (Fig. 5). The LUMO of the monoHER ortho-quinone and the quercetin quinone methide are 42.6 kJ/mol and 0.0605 kJ/mol, respectively, showing that oxidized monoHER is a harder electrophile than oxidized quercetin. The HOMO of ascorbate and GSH are −394 kJ/mol and −1.35 kJ/mol, respectively, showing that ascorbate is a harder nucleophile than GSH. According to Pearson's HSAB concept [28], hard electrophiles react faster and form stronger bonds with hard nucleophiles, explaining the preferential reaction of oxidized monoHER with ascorbate over thiols.

## Discussion

Paradoxically, free radical scavenging antioxidants are chemically converted into potentially harmful oxidation products when they protect against free radicals [29]. These oxidation products usually retain a part of the reactivity of the species they have scavenged, and might therefore cause damage to vital cellular targets [19], [30]. To protect cells against this damage the human body has a refined network in which the reactivity is transferred from one antioxidant to another, thereby gradually diminishing the reactivity [1], [29].

The aim of the present study was to investigate how monoHER fits into this endogenous antioxidant network. The interaction of monoHER with the network was compared with that of quercetin, which chemically closely resembles monoHER. The results of this study show that oxidized monoHER is reduced by ascorbate to recycle the parent compound monoHER, while oxidized monoHER reacts with GSH to form a GSH-conjugate. The reactions of oxidized quercetin with ascorbate and GSH are similar to those of oxidized monoHER [26]. However, as shown in the present study, a major difference is that oxidized quercetin preferentially reacts with thiols, whereas oxidized monoHER preferentially reacts with ascorbate. This is an essential difference in the interplay of both flavonoids with antioxidants of the endogenous antioxidant network.

The different position of monoHER and quercetin in the network has to originate from an intrinsic difference in the chemical nature of their oxidation products. Quantum chemical calculations revealed that of the four possible tautomeric forms of oxidized quercetin, the tautomer shown in Fig. 5A, has an abundance of more than 99%. In this tautomer the distance between the electron deficient carbonyl centers is maximal, which is energetically favorable and explains its high abundance. The high abundance of this specific tautomer is corroborated by the formation of adducts in the A ring, i.e. 6-GSH-quercetin and 8-GSH-quercetin, in the reaction of GSH with oxidized quercetin [31].

In monoHER a rutinose is attached to the 3-OH group of the C ring and a hydroxyethyl group is attached to the hydroxyl group oxygen at position 7 of the A ring. These substitutions preclude the formation of quinone methide tautomers in oxidized monoHER. Therefore, only the ortho-quinone can be formed (Fig. 5B). In this ortho-quinone two carbonyls are adjacent, which is energetically unfavorable compared to the larger distance between these groups in the preferential tautomer of oxidized quercetin. The presence of an ortho-quinone in the B ring is corroborated by the formation of an adduct in this ring, i.e. 2′-GSH-monoHER, in the reaction of oxidized monoHER with GSH [25].

Apparently, the oxidation products of monoHER and quercetin are energetically different. The LUMO of oxidized monoHER is primarily concentrated in the B ring and therefore relatively high, while that of oxidized quercetin is spread over the whole molecule (Fig. 5). This is reflected by a LUMO of oxidized quercetin (0.0605 kJ/mol) that is substantially lower than that of oxidized monoHER (42.6 kJ/mol). Pearson's HSAB concept assigns the terms ‘hard’ or ‘soft’ to chemical species to explain or predict the outcome of a chemical reaction [28]. ‘Hard’ applies to electrophiles (the reactants that accept binding electrons) that have LUMO of high energy or nucleophiles (the reactants that donate binding electrons) with a low HOMO energy. ‘Soft’, on the other hand, applies to electrophiles with a low LUMO value or nucleophiles with a high HOMO value. According to the HSAB concept, hard electrophiles react faster and form stronger bonds with hard nucleophiles, whereas soft electrophiles react faster and from stronger bonds with soft nucleophiles.

**Figure 6:**
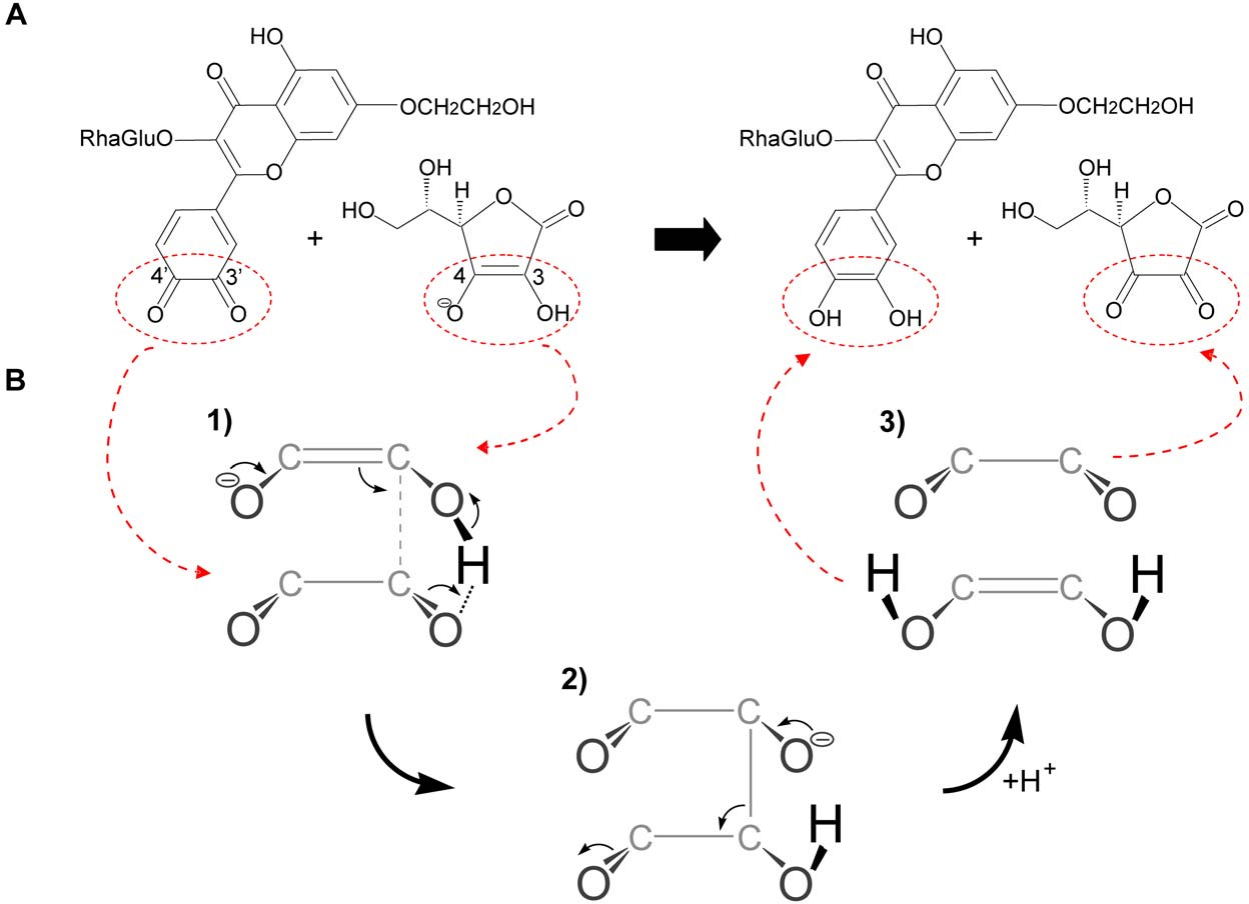
Chemical reaction of oxidized monoHER with ascorbate. (A) Chemical structure of oxidized monoHER (left) and ascorbate (right). The active part of ascorbate and the active part of oxidized monoHER are indicated by the red ellipses. (B) Suggested route for the reaction of oxidized monoHER with ascorbate. Only the active parts are shown to illustrate the suggested mechanism more clearly. (1) The active part of ascorbate (top) approaches the active part of oxidized monoHER (bottom) due to a π-π interaction and a hydrogen bond. The π-electrons of ascorbate are used to create a new bond. The C3 of ascorbate will most likely attack the C3′ of the monoHER quinone because it is more electron deficient than the C4′ according to Spartan ‘06. (2) After the attack, a transition state, with an *sp3* bond between ascorbate and oxidized monoHER, is suggested to be formed. (3) This intermediate rapidly decomposes into monoHER and oxidized ascorbate. The driving force of this reaction is the restoration of the highly conjugated π-system of monoHER.

Based on their LUMO values, oxidized quercetin is a softer electrophile than oxidized monoHER. The reaction of GSH with both oxidized monoHER and quercetin is a Michael addition in which GSH acts as a nucleophile. The reaction with ascorbate is a redox reaction in which ascorbate finally donates two electrons to the oxidized products. GSH is a relatively soft nucleophile (HOMO value of −1.35 kJ/mol) compared to ascorbate (HOMO value of −394 kJ/mol). This can explain the preferential reaction of the soft electrophile, oxidized quercetin, with thiols over ascorbate. Oxidized monoHER, on the other hand, is a harder electrophile than oxidized quercetin explaining its preference for the harder nucleophile ascorbate over GSH. Moreover, as depicted in Fig. 6A, the active part of ascorbate can approach the active part of oxidized monoHER by a hydrogen bond and a π-π interaction between ascorbate and the ortho-quinone. The reaction between oxidized monoHER and ascorbate is presented step by step in Fig. 6B.

**Figure 7:**
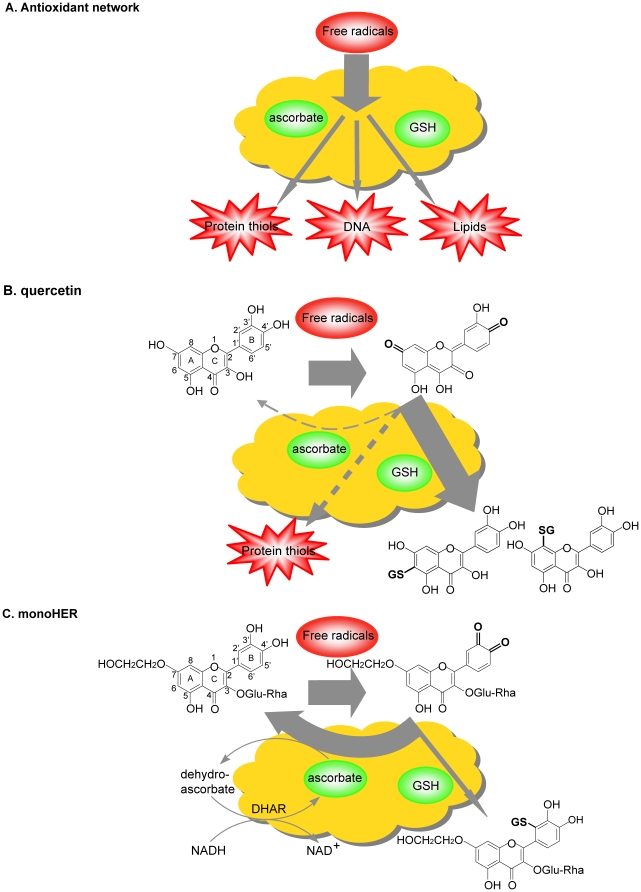
Interplay of monoHER and quercetin with the endogenous antioxidant network. (A) Schematic representation of the endogenous antioxidant network. Free radicals are scavenged by antioxidants in the network, such as GSH and ascorbate. In this way, free radicals are neutralized. Free radicals that are not neutralized can damage e.g. proteins, lipids and DNA. (B) The flavonoid quercetin is an excellent radical scavenger. During the scavenging of free radicals quercetin becomes oxidized. After oxidation of quercetin, four tautomeric forms of the oxidation product can be formed. In the figure the tautomer which has an abundance of more than 99% is shown. When ascorbate and GSH are present in the same concentration, oxidized quercetin reacts much faster with GSH than with ascorbate, thereby forming 6-GSH-quercetin and 8-GSH-quercetin. Because of its high reactivity towards thiols, oxidized quercetin is also prone to react with protein thiols, as was seen in human blood plasma. This reaction of oxidized quercetin is not prevented by ascorbate and can lead to toxicity. (C) The oxidation product formed out of monoHER is an ortho-quinone. Ascorbate recycles this oxidation product to the parent compound monoHER, while GSH forms a conjugate with oxidized monoHER, i.e. 2′-GSH-monoHER. When both compounds are present in the same concentration, oxidized monoHER reacts rather with ascorbate (73%) than with GSH (27%). The oxidized ascorbate formed in this recycling can be regenerated in the network, e.g. by dehydroascorbate reductase (DHAR) that uses NADH as cofactor. Thus, the advantage of monoHER is that it can safely channel the non-specific reactivity of radicals toward ascorbate, which can be regenerated in the antioxidant network.

Based on our findings, the following concept is proposed. Flavonoids easily pick up the reactivity of radicals due to their superior scavenging activity. This reactivity is directed in different ways by the two flavonoids studied (Fig. 7). Quercetin directs this reactivity towards thiols. Conjugation of oxidized quercetin with GSH is primarily a cellular defense mechanism to alleviate the harmful consequences of the reactive quinone metabolite [32]. However, this will reduce GSH levels and thus weakens the endogenous antioxidant network. Moreover, in e.g. blood plasma, where GSH is practically absent, or when GSH has been depleted, oxidized quercetin will react with protein thiols. This causes toxicity such as increased membrane permeability [33] or impaired functioning of enzymes that contain a critical thiol-group [34], [35]. In contrast to quercetin, monoHER preferentially directs its acquired reactivity towards ascorbate. In human blood plasma, oxidized monoHER, contrary to oxidized quercetin, does not react with plasma protein thiols. Ascorbate present in plasma reduces oxidized monoHER to the parent compound and prevents that oxidized monoHER reacts with thiols. The oxidized ascorbate formed in this recycling of monoHER can also be regenerated in the network, e.g. by dehydroascorbate reductase that uses NADH as cofactor. In this way, the reactivity is completely neutralized and the antioxidant network is restored. Thus, the advantage of monoHER is that it can function as a catalyst that safely channels the reactivity of radicals into the endogenous antioxidant network. This advantage might have been involved in the superior effect of monoHER over other structurally related flavonoids [36] in our screening procedure for protection against doxorubicin-induced cardiotoxicity. To conclude, our study demonstrates that structurally related flavonoids, belonging to the same subgroup and displaying a comparable radical scavenging activity, can have a different impact on health.
